# Strategies for the Global Eradication of Peste des Petits Ruminants: An Argument for the Use of Guerrilla Rather Than Trench Warfare

**DOI:** 10.3389/fvets.2019.00331

**Published:** 2019-09-26

**Authors:** Angus R. Cameron

**Affiliations:** Ausvet Europe, Lyon, France

**Keywords:** PPR, disease eradication, strategic vaccination, movement management, surveillance, user-focused

## Abstract

Many historical disease eradication campaigns have been characterized by large-scale mobilization and long-term campaigns of mass vaccination. As the duration of a program increases, the total cost also increases, but the effectiveness and sustainability decrease, sometimes resulting in premature loss of stakeholder support, field team fatigue, and failure or major set-backs. In contrast to this trench warfare approach, this paper proposes an eradication strategy modeled on guerrilla tactics: use exceptionally good, locally relevant and timely intelligence; strike rapidly and effectively in small areas; achieve your goals; and keep moving. For peste des petits ruminants eradication, this means a shift away from long-term mass vaccination, focusing instead on addressing some of the challenges that have plagued previous eradication programs: ineffective surveillance and movement management. Recent developments in surveillance have shown that it is now feasible to capture information about almost all cases of disease, all movements and all control activities, from the entire population in real time. Developing powerful, effective and sustainable surveillance systems is an essential prerequisite for rapid, affordable PPR eradication. PPR can be rapidly eliminated from small populations by achieving very high levels of vaccination coverage for only a short period. The key challenge is then to prevent the re-introduction of disease as immunity wanes, and to respond rapidly and effectively in the case of further local outbreaks. A comprehensive understanding of movement patterns and their drivers will allow rapid progressive eradication to be implemented. The population can be divided into manageably small units, targeted sequentially for high-coverage short-duration vaccination, then moving to the next unit based on the distribution of disease and the direction of animal flow. This approach optimizes the use of available resources, and minimizes the challenge and disruption of managing retrograde movement from infected to uninfected areas. High levels of community engagement are required to achieve the quality of surveillance, movement management and rapid response necessary for success. Traditionally, long-term vaccination has been used to first eliminate the virus from a population, and then to protect it against re-introduction of the disease. Under the guerrilla strategy, continuous real-time information, not long-term vaccination, is the main tool for disease eradication.

## Introduction

Following the declaration of successful global eradication of rinderpest, peste des petits ruminants (PPR) has been proposed as a candidate for global eradication (1). The World Organization for Animal Health (OIE) and the Food and Agriculture of the United Nations (FAO) released a strategy in 2015 aiming for global eradication of PPR by 2030 (2). Jones et al. (3) have developed and assessed an alternative strategy for eradication, one of the features of which is time-bound vaccination to avoid the need for long-term costly control programs (trench warfare). This paper builds on the detailed work already undertaken, incorporating recent experience in sociological approaches to user-focused surveillance (4), and a consideration of disease control theory, to present a more aggressive (guerrilla) strategy for rapid, affordable global PPR eradication. Global disease eradication is enormously complex, and requires many components. This paper focuses on specific technical areas that differ from those already developed, building on previous work.

### Factors Influencing Likelihood of Eradication

In order to survive and reproduce, viruses like PPR need to be transmitted from one host to another. Control and eradication strategies are focused on interrupting transmission. The feasibility of this depends on the characteristics of the virus and the populations that it infects. A number of factors support PPR eradication:

- **Survival of the virus outside the host**. PPR is fragile outside the host as its lipid bilayer envelope is rapidly destroyed by heat and sunlight (5, 6). It is therefore mainly transmitted by direct contact (bodily secretions), local aerosol spread from coughing, or contaminated feed or water, but only to animals within close proximity.
- **Vaccine**. The currently used homologous attenuated PPR vaccine has major advantages: it protects against all lineages; it provides long lasting protection (at least 3–5 years, but probably life-long); it is safe, in that it has not reverted to virulence and does not cause abortion; and it is widely available and quality controlled (6).
- **Hosts range**. There is no prolonged carrier state after infection, and there are no known reservoirs outside domestic small ruminants (or at least none that are likely to play an epidemiologically significant role) (6).
- **Diagnosis**. Many cases demonstrate evident clinical signs that are easily detected by herders. In previously free populations, the disease takes an epidemic form, with high morbidity and mortality and acute clinical expression, making clinical detection relatively reliable. There are good laboratory and field-based diagnostic tests available.

**Figure 1:**
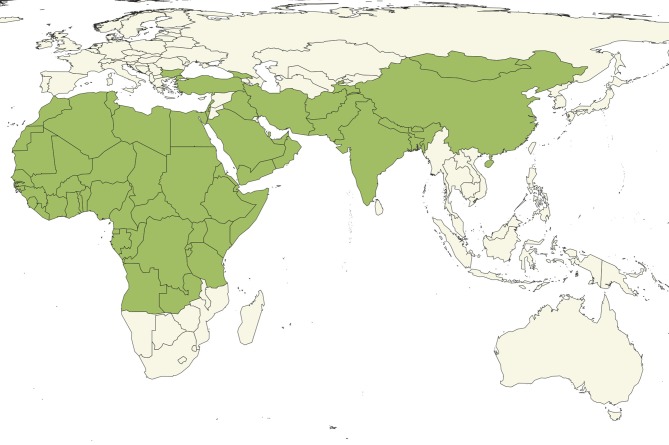
Countries (green) reporting the presence of PPR to OIE as of April 2019 (8).

On the other hand, there are a number of potential constraints:

- **Hosts**. There are still questions about the role of some other species in the epidemiology of PPR, including dromedaries, wildlife and bovines (7).
- **Distribution**. PPR is extremely widespread (Figure 1) and endemic in many countries with under-resourced veterinary services.
- **Population dynamics**. Small ruminants have a high population turnover, resulting in the rapid introduction of naïve animals into vaccinated populations (6). Local animal density within flocks is high, facilitating rapid within-flock spread. In many endemic areas, farming practices include transhumance and migratory management, increasing the opportunity for disease spread.
- **Economics**. While the total cost of disease is high, the value of individual animals (relative to cattle, for example) is much lower. This, coupled with short lifespan means that the proportional cost of vaccination is higher than was the case for rinderpest.
- **Clinical expression**. Expression varies with species and breed, and the signs are not specific making a definitive clinical diagnosis difficult or impossible. In endemic areas, virus may circulate with little clinical expression.

### Proposed Strategy

This paper presents a hypothesis: that by learning from the lessons of rinderpest, building on existing ideas (2, 3, 6) and epidemiological theory, and incorporating new technological, sociological and epidemiological developments, a new approach to PPR eradication is possible—one that will be able to achieve global eradication more rapidly, less expensively and with longer term sustainable benefits than traditional approaches. The key elements of this new approach, to be expanded upon in the following sections, are:

- Aiming for **rapid global eradication**—to avoid donor and veterinary service fatigue
- Achieving effective **global coordination**—focusing disease eradication efforts on the disease, populations, ecosystems, animal movement, and other risk factors, not on national boundaries or government administrations
- Progressive eradication by dividing the population into **small units**—allowing intensive allocation of resources in a small area, to achieve very high vaccination coverage
- Using very **short-duration local control interventions**—to avoid eroding the support of producers and other local stakeholders
- Intelligent focused **movement management**, and carefully sequenced spatial and temporal progression of eradication activities—to minimize the disruption to producers and markets and avoid introducing price distortions that provide incentives for dangerous movements
- Aiming for a short period of very high vaccination coverage within each population unit to achieve virus elimination and then quickly return to a largely susceptible population—to **maximize the sensitivity of clinical surveillance** for detecting new outbreaks
- Maintain high levels of **producer engagement** in disease surveillance and control, along with rapid, effective outbreak response capacity—to detect and rapidly eliminate new outbreaks in otherwise free areas
- Underpin everything with **excellent information**: sustainable, affordable, real-time, census-level, highly granular, and integrated surveillance covering all aspects of the eradication program, including animal movements and their drivers, population, vaccination, veterinary infrastructure and resources available, control activities, disease occurrence, and outbreak response.

This hypothesis has been developed on the basis of epidemiological theory and evidence from previous eradication programs. While other authors (9) have emphasized the need for more strategic vaccination based on better surveillance, this guerrilla approach to small-area intensive vaccination, and rapid planned progression through population units may be perceived as carrying higher risks. These are mitigated by a greatly increased emphasis on high quality information generated using existing approaches to effective stakeholder engagement.

The purpose of a hypothesis is to be tested, and this is often achieved by small-scale experimentation. Unfortunately, testing this hypothesis will require large scale investment and commitment, ideally at a regional level. This paper is intended to start a conversation as to whether the hypothesis has enough merit to warrant such a large-scale test.

## Lessons Learned

### What Can We Learn From Rinderpest Eradication?

The global eradication of Rinderpest was announced in 2011 and represents a landmark for livestock disease control (10). The first major coordinated rinderpest eradication program was the 15-country Joint Program (JP15), launched in Africa in 1961. It went through several stages of evolution [Pan-African Rinderpest Campaign (PARC), The Pan African Program for the Control of Epizootics (PACE), and the Global Rinderpest Eradication Program (GREP)] and took 50 years for eradication to succeed. Without taking away from this remarkable success, it is important to ask, as we consider embarking on another livestock morbillivirus eradication program, whether we can do it better—faster, more cost-effectively, and with even greater net benefit.

The expected duration of the eradication program is perhaps the most critical factor influencing cost. It is extremely unlikely that even the most visionary donor or finance department would willingly embark on a program, knowing that it may not succeed for 50 years. Learning the lessons from rinderpest eradication may help achieve the goal more quickly, at low cost, and with greater confidence (11).

#### Withdrawing Support too Early

In 1979 after 18 years, JP15 had successfully decreased rinderpest in participating countries to very low levels, with only a few sporadic outbreaks. Unfortunately, the disease fought back, with extensive spread in five of the participating countries. In 1986, PARC was initiated, but faced a far greater challenge, and took 15 more years to succeed.

#### Lack of Access to, or Use of, Epidemiological Information

In 1999, the Intensified GREP program changed the approach to control by improving the use of surveillance information to focus on localized reservoirs of infection. This approach was first developed in Ethiopia and achieved considerable success. It was then extended to Sudan, the Arabian Peninsula, Pakistan and East Africa resulting in rapid eradication by 2001 (10).

One of the summary conclusions of the 2010 GREP symposium (11) was that “newer approaches such as immunosterilization and community-based vaccine delivery with heat-tolerant vaccine … made a valuable contribution in South Sudan. Noting that future control campaigns against PPR may require even more vaccination than did rinderpest, several participants advocated the use of more modern approaches from the start and suggest that additional innovative thinking for epidemiological targeting and vaccine delivery may be necessary.”

#### Lack of Community Engagement

“Another lesson was that there must be communication with cattle keepers to convince them of the need for vaccination and counter other considerations that could argue against them having their animals vaccinated. As a result of not taking these and other considerations into account, JP15 controlled rinderpest but did not eradicate it, and the disease returned as a major epidemic in Africa” (9). It could be contended that our understanding of community engagement has evolved since these words were written. We should no longer seek to “convince” farmers of the need for vaccination, but instead to engage them as full partners in disease eradication, placing their needs at the center of the program (4).

#### Ineffective Vaccination Coverage

At times during rinderpest eradication, sufficient levels of population immunity were not being achieved to attain eradication. Indeed, it was proposed that sub-optimal vaccination could mask the presence of disease and decrease the efficacy of surveillance programs, and could be worse than no vaccination (12). In India, mass vaccination was only able to achieve coverage rates of 40–50% (13).

#### Mass Vaccination Strategy

“An important lesson from JP15 was that 3 years of blanket vaccination with no regard for the epidemiological significance of cattle numbers, distributions, movements and husbandry was not an appropriate strategy” (9). Taylor et al. (14) noted the success of so-called immunosterilization, which they defined as two doses within 6 months designed to eradicate disease from the population. They further noted that “… in an immunosterilization campaign the critical issue was to disrupt viral transmission through the short-term generation of a highly immunized population. Relative to the desired objective, it did not particularly matter if, in the succeeding months, the population remained cohesive and highly immune, or fragmentary and increasingly susceptible, provided that at the time a serviceable herd immunity had been generated” (15).

#### Inadequate Coordination

Imperfect local and international coordination hampered progress. This is linked to inadequate donor coordination and commitment.

#### Lack of Broad and Sustainable Benefit

“It could help motivation and prioritization in developing countries if future programmes move from the control of a single disease to a broader remit. The control of livestock diseases that affect trade, including livestock exports, may encourage developing countries' participation. Mechanisms need to be found for sustained support for surveillance, diagnosis and response to trade-related diseases and emerging infectious diseases, including zoonoses” (16).

#### Institutionalization

A (possibly theoretical) challenge to rapid and effective disease control is the process of institutionalization. A major and prolonged eradication project brings with it organizational infrastructure—offices, personnel, equipment—that carry their own inertia. In particular, when a person's employment is directly linked to the eradication of a disease, the successful completion of that task necessarily raises the likelihood of termination of the position, especially if it is funded through external sources. This may represent a conflict of interest—the act of working toward eradication is more lucrative for individuals than achieving it.

### Conclusions

Based on the experience of rinderpest, a major constraint to successful eradication is the duration of the eradication campaign. As a campaign drags out:

- The cumulative cost mounts, decreasing the appetite of governments or donors to continue to contribute.
- Operational fatigue sets in, affecting both farmers and field personnel. The initial enthusiasm to pull together to fight a common enemy erodes until the work becomes routine and apparently endless. Vaccination coverage levels drop and the quality of surveillance deteriorates.
- Political will wanes, as other competing priorities arise, risking premature termination of the program, especially during the final stages when progress is harder to measure but costs remain high.
- Control activities risk becoming institutionalized, and lose flexibility and responsiveness.

The other main constraint has been information. While rinderpest eradication led to some major developments in surveillance methodologies, including participatory epidemiology (17), most of the program (until only a few years before final successful eradication) was hampered by a lack of comprehensive understanding of the populations at risk, movement patterns, early detection capacity, and accurate measures of vaccination coverage. This was exacerbated by weakness in disease information systems.

### Key Objective

Based on the lessons from rinderpest, it is possible to identify some simple key objectives for a future PPR eradication program: rapid eradication, based on the effective use of good information. This is likely to require a relatively high, shorter term investment, but will be able to maintain greater motivation and higher efficiency.

## Disease Eradication Theory

This discussion presents a simplified consideration of the theoretical basis for disease eradication, building on the principle already introduced (18): to persist, viruses need access to new hosts, which may be introduced to a population by movement or birth. Preventing access can be achieved through two main methods: vaccination or movement management.

### Vaccination for Disease Eradication

Consider a hypothetical virus that is transmitted only by direct contact; for which there is an effective vaccine that provides life-long immunity in 100% of vaccinated animals; there is no carrier state or wildlife reservoirs; and infected animals either die or recover after which they are rapidly free from virus (within 4 weeks) and have persistent immunity. The areas in which PPRV differs from this hypothetical virus will be discussed below.

In a closed population in which infected animals are present, vaccination which generates immunity of all animals will result in rapid elimination of the virus. Infected animals die or recover, and no new animals are able to be infected. A single round of vaccination with 100% coverage should be adequate (14, 15). Why then is it so hard to eradicate disease? The answer lies in the realities of vaccination programs and population dynamics.

- **Vaccination coverage**: it is often very difficult to use vaccination to protect 100% of animals in an area. This may be related to communication with owners or herders, difficult access, lack of owner compliance, inadequate vaccine, inadequate time or human resources, or corruption (on-selling or discarding vaccine and falsely reporting that vaccination has been completed).
- **Rate of vaccination**: in large populations, all animals cannot be vaccinated simultaneously. By the end of a vaccination round, immunity in part of the population may be falling (see section Timing Considerations) or non-immune animals may be introduced.
- **Immune response**: vaccinated animals don't necessarily develop immunity to the field virus. This may be due to inappropriate choice of vaccine (unlikely to be a problem for PPR), poor vaccine quality control and potency, suboptimal handling of the vaccine during storage and transport resulting in decreased efficacy, poor vaccination technique resulting in failure to deliver an adequate dose in the right location, or poor immune response within the animal due, for example, to stress, poor nutrition, concurrent disease, or interference with maternal immunity in the offspring of seropositive animals,.
- **Reproduction**: populations are not closed and turnover is rapid. Depending on seasonal lambing/kidding patterns, a high proportion of the population may be replaced with non-immune animals in a short period.
- **Animal movements**: even if movements from an infected area are blocked, movements from free areas may result in the introduction of non-immune animals, diluting the proportion of protected animals.

This means that achieving a population that is 100% immune for long enough for the disease to be eradicated is difficult. In the past, control and eradication programs, for both rinderpest and PPR (15) have acknowledged these constraints and overcome them with longer periods of protection. Instead, the guerrilla strategy seeks to strategically address these constraints to achieve very high levels of protection for a short period.

#### Herd Immunity

The constraints identified above are normally partly addressed by the concept of herd immunity. The effective reproductive rate (R) is a measure of the average number of new cases of disease generated by an infected animal (19) in a partially immune population. If R is <1, the disease will, over time, die out. Herd immunity is achieved when the proportion of vaccinated animals is high enough to decrease R to below one.

Many disease control programs focus on estimating the vaccination coverage required to maintain R below one [for example, the Global Strategy targets an immunity of between 70 and 80% (2)] and aim to maintain that level of coverage for a prolonged period. This is because the time required for the disease to be eradicated due to herd immunity is influenced by R and the population size.

If R = 1, the disease will maintain itself at a steady state. As R decreases due to higher vaccination rates, the time to elimination of the disease becomes shorter. At the limit, if R = 0 (100% effective vaccination), the disease will be eliminated in the space of a single infectious period (plus the duration of survival of the agent in the environment). In very small populations, elimination is faster due to integer mathematics effects. With a population of 10, when prevalence falls below 10% (a single animal) the disease must be eradicated. With a population of 10,000, the prevalence needs to be below 0.01% for eradication.

#### Population Size

**Figure 2:**
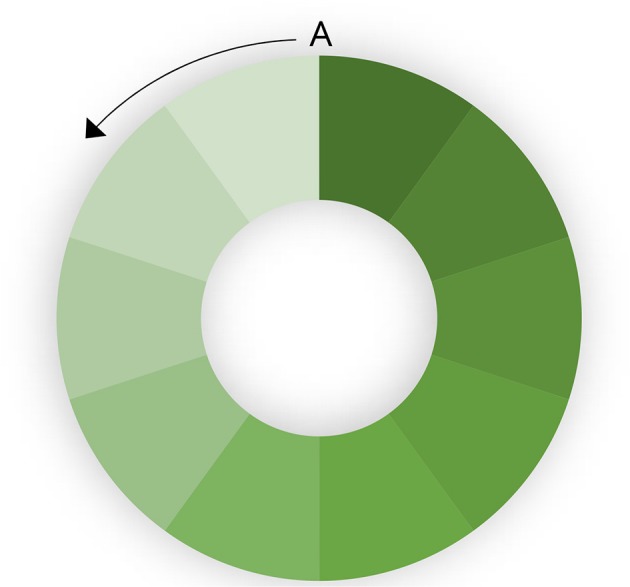
Effect of progressive vaccination in a large population. The color intensity indicates population immunity. Vaccination starts at point A and progresses anticlockwise.

The rate at which a population is vaccinated also has an impact on the ability to eradicate disease. If the entire population is vaccinated simultaneously, eradication will be faster. If there is progressive vaccination, population turnover (loss of immune animals through slaughter) and the introduction of new susceptible animals (through birth or introduction) will decrease herd immunity in part of the population, leading to heterogenous R. This is illustrated in a hypothetical population in Figure 2. A circular population is used to clarify the effect. If vaccination is started at point A and progresses in an anticlockwise direction, population turnover will mean that the vaccination coverage (indicated by depth of shading) in the first vaccinated part of the population is relatively low by the time the last part of the population is vaccinated. This means that there is a risk that infected shedding animals in the last (unvaccinated) part of the population are in contact with the first vaccinated part of the population in which immunity is decreasing. If the rate of vaccination is too slow, and the population turnover too high, it may still be possible to maintain the virus in the population despite ongoing high-coverage vaccination at too slow a rate.

The solution to these problems is to keep the population small. By dividing the population into small population units (for example a large flock, a village or a subdistrict), it is possible to achieve near simultaneous vaccination of the whole population unit. It is also much more feasible to achieve near 100% coverage of the population, by applying available resources more intensively to a smaller population. The conclusion is that viral elimination through vaccination can be achieved more reliably and more quickly if the population is divided into relatively small subunits. The size of the population unit should be small enough to achieve close to 100% coverage, rapidly enough to maintain near complete protection before non-immune animals enter the population (for example, through lambing or kidding).

#### Timing Considerations

A population unit should be considered free from PPRV when all animals have been vaccinated (in such a way as to provide the greatest chance for a very high immune coverage), and enough time has passed such that protective antibodies have developed and any animal infected at the time of the vaccination has either died or recovered, and is no longer at risk of shedding virus.

Protective antibodies develop within 1 week of vaccination (20). There is scant information on the period of viral shedding after infection. Parida et al. (21) found that 14 days after infection (the last sampling date), 6 out of 10 nasal swabs from infected goats were PCR positive, while only 1 and 0 were positive from saliva and eye swabs, respectively. Lui et al. (22) found PCR positive ocular secretions in one of 12 goats, 26 days after infection (in a study that ran for 40 days). In both studies, the presence of viral RNA does not necessarily mean that viable virus is being shed. In the absence of transmission studies, and on the basis of the available evidence, it would seem prudent to assume that virus may be shed for up to 4 weeks after infection.

Based on this assessment, population units in which 100% of animals are effectively vaccinated may be considered free from infection 5 weeks later, assuming a high proportion of animals develop immunity following vaccination.

The timing of vaccination also plays a role. Vaccination should be avoided shortly before lambing/kidding, to avoid a rapid decrease in the proportion of immune animals in the population unit as a result of dilution of the immune adults by a large influx of non-immune lambs or kids. It should be timed to avoid times of peak demand for animal movement (see section Theory of Animal Movement Management) in order to allow the population to be closed during the period of virus eradication. Periods of migration or when flocks are inaccessible due to remote grazing areas should be avoided to overcome problems of access and to facilitate high vaccination rates. It should also be undertaken at a time when animals are at their healthiest (e.g., have access to good nutrition) to maximize the chance of developing protective immunity in response to vaccination. It is likely that some of these conditions may be contradictory, and compromises need to be made, or that pauses in the vaccination program will be required at certain times of year. A detailed understanding of populations, reproductive patterns, husbandry and movements are required to plan the optimal vaccination strategy in specific environments. Modeling approaches have been used to address this challenge (23, 24).

### Theory of Animal Movement Management

As with vaccination, it is theoretically possible that movement management alone might be used to eradicate the virus. Assume that the population is divided into small closed units such that all animals are in frequent contact with all others within the unit, and that all movement between units is prevented, so there can be no introduction of infection into uninfected units. Under this scenario, the virus would die out in all infected units, because all susceptible animals in infected units rapidly acquire natural immunity (or die). None of the uninfected units would become infected. Based on this approach, the entire population could become free from infection, within the time it takes for susceptible animals within infected units to become infected and immune (or die).

This theoretical approach is not feasible: it is difficult or impossible to impose a complete movement restriction on the entire population for long enough for the disease to die out; and there are other means of spread of the virus, such as fomites.

The reason that movement restrictions are difficult to implement and to maintain is because livestock production is based on the need for movement. Markets (demand) are generally located in different areas to production (supply); in many production systems, access to feed requires frequent, constant or seasonal movements; and in some systems, breeding is achieved by the movement of males from flock to flock.

When there is an economic imperative to move animals, imposing movement restrictions results in economic hardship for producers, as well as distorting the market, providing a strong financial incentive to circumvent restrictions. If movement from a production area to a market area is prohibited, the price differential between the two will increase, providing a strong motivation for illegal activity, especially when a family's main source of income may be at risk. Similarly, if movement for grazing is prohibited, the animals' survival may depend on illegal movement.

The challenge is therefore to understand how to prevent the spread of disease through animal movements, while avoiding distorting markets. The solution to this problem is to reduce movement restrictions to the very minimum required to prevent the spread of disease from infected to known uninfected population units, while allowing enough safe movements to avoid distorting the market.

#### Optimizing the Eradication Sequence

The role of animal movement networks and population sizes in disease control and extinction has been extensively studied (25–28) and this work provides a basis for detailed analysis and modeling to optimize the size of populations for disease control and the sequence of eradication. This discussion provides a simple overview of the approach, to illustrate its value in PPR eradication. Rather than impose a complete movement standstill, it is important to recognize that a large proportion of routine movements pose no significant threat to successful eradication. Population units may be classified as infected (I), and not yet part of an active eradication program, known to be free (F), either historically or due to successful eradication, or the subject of active control or eradication (C) efforts.

Table 1 shows typical rules for animal movements between units of different statuses.

**Table 1:** Rules for movements between units of different status.

**Origin**	**Destination**		
	**I**	**C**	**F**
**Infected**	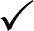	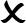	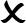
**Control**	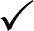	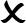	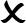
**Free**	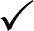	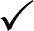 ^a^	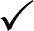

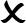
: movement not allowed; 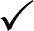: movement allowed.

a *Susceptible animals moving from free to control population units risk diluting the population immunity. Only animals known to be immune should be allowed to enter active control units*.

Different population units have different movement patterns. In a breeding area, the bulk of movements are outgoing (with strong seasonal variation), while in a consumption area (with an abattoir, for example), the bulk of movements are incoming. A fattening area may have roughly equal incoming and outgoing movements, but during different seasons. A market area may have balanced inward and outward movements on a daily basis.

**Figure 3:**
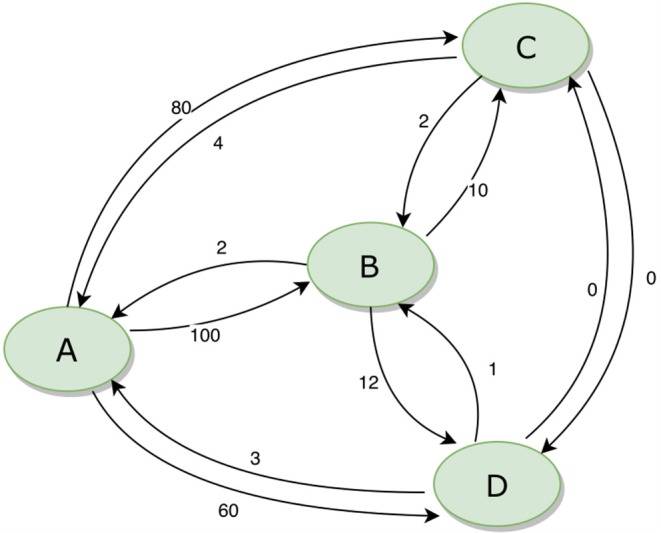
Example of a simple animal movement network diagram for four population units. The numbers beside each arrow indicate the number of animals moving between the population units.

The movement of animals between units can be expressed as a network. Figure 3 provides an example of a simple network of four population units, with movements in and out (for any reason, including trade, transhumance, etc.) indicated. During disease eradication, implementation of movement restrictions will block movements between some units depending on their different statuses. The sequence in which disease eradication is carried out in the population units can have a major impact on the total number of movements that need to be restricted, as shown in Table 2.

**Table 2:** Total number of blocked movements required during an eradication program, based on all possible sequences of eradication in four population units (A, B, C, and D) as illustrated in Figure 3.

**Sequence**	**Movements**	**Sequence**	**Movements**	**Sequence**	**Movements**	**Sequence**	**Movements**
ABCD	35	DABC	176	DBAC	418	DBCA	728
ABDC	37	DACB	202	DCAB	422	DCBA	736
ACBD	48	CABD	205	BDAC	438	BDCA	748
ADBC	56	BACD	227	CDAB	440	CDBA	754
ACDB	79	BADC	229	CBAD	469	CBDA	762
ADCB	82	CADB	236	BCAD	474	BCDA	767

If eradication starts in a breeding area (A), when most movements are outgoing, there will be minimal disruption to the market. In contrast, if eradication starts in a consumption area (B or C), many movements will need to be blocked, risking major disruption and making it much more difficult to successfully implement effective movement management. The best option (ABCD) results in only 5% of the blocked movements of the worst option (BCDA).

#### Real World Examples

With a small number of nodes such as those used in this example, the optimal permutation can be calculated manually. For a large number of nodes, and for seasonally varying movement patterns, the challenge is much more significant. Two examples were used to illustrate this using real world movement data: goat movements on Java Island in Indonesia, and cattle and deer movements in New Zealand. Neither area is infected with PPR but are used because of the availability of high quality, contrasting movement data.

##### Goat Movements in Java, Indonesia (2016)

In Indonesia, the iSIKHNAS animal health and production information system provides a practical source of detailed individual animal movements which can be used to simulate and evaluate alternative sequences of control activities (29). Data on all recorded individual goat movements on the island of Java in 2016 was extracted from the iSIKHNAS database, as an origin-destination matrix. For the purposes of this example, the district (kabupaten) is used as the population unit, although smaller units would be likely to be used in practice. Of the 119 districts in Java, 60 recorded intra-island goat movements in or out during 2016, with a total of 54,995 animals moved.

**Figure 4:**
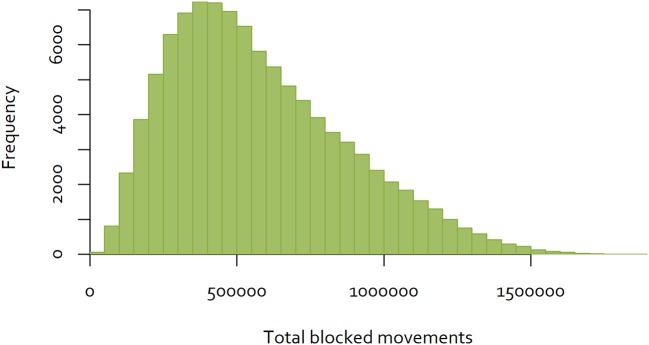
Distribution of the number of blocked movements for goats on Java, over 100,000 random sequences of control.

An exhaustive analysis of all combinations of the 60 districts would require analysis of 8.3 × 10^81^ combinations. Dynamic programming techniques may provide a feasible approach to finding the optimal combination (30). However, for the purposes of this analysis, a simple analytical tool was developed using R (31), to calculate the total number of blocked movements based on the Java goat movement data, for a given sequence of eradication over the 60 districts. A sample of eradication sequences was generated by simulating 100,000 random sequences, and calculating total blocked movements for each. The distribution of the results is shown in Figure 4.

**Figure 5:**
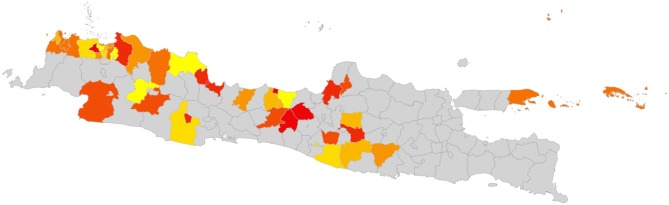
Best identified sequence of control for goats on Java island, to minimize disruption of animal movements. Red indicates the first districts, yellow the last districts in the sequence.

While this approach is not able to determine the single optimal sequence of eradication to minimize disruption, selecting the sequence with the minimum blocked movements will provide a “good” option. In the simulation illustrated above, the best sequence resulted in 17,279 blocked movements over the 60 eradication time periods (Figure 5), while the worst resulted in 1,896,292 blocked movements. While better and worse sequences are likely to exist, the use of the best simulated sequence would result in 0.9% of blocked movements relative to the worst. This is an example of a low-density matrix, where only 4.8% of cells had a movement recorded. More importantly, the Java goat matrix is very asymmetrical—only 0.19% of district pairs had reciprocal movements.

##### Cattle and deer movements in New Zealand (2018)

**Figure 6:**
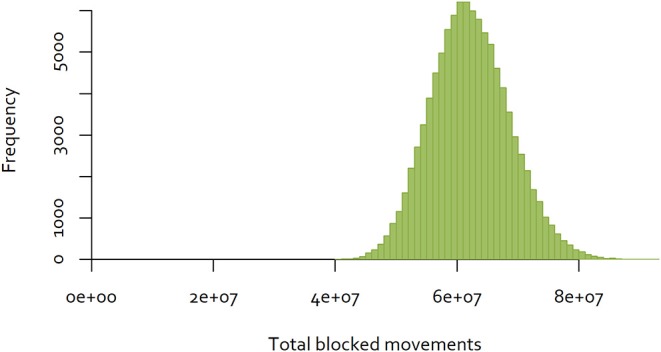
Distribution of the number of blocked movements in New Zealand, over 100,000 random sequences of control.

The same approach was used to a dataset consisting of all completed cattle and deer movements in New Zealand in 2018, consisting of movements between 73 cities and districts (32). In contrast to the Java data, 40.98% of movements were reciprocal. The distribution of the results for 100,000 random sequences are illustrated in Figure 6. In this case, the minimum number of blocked movements calculated was 40,392,916, which is 43% of the largest number observed.

**Figure 7:**
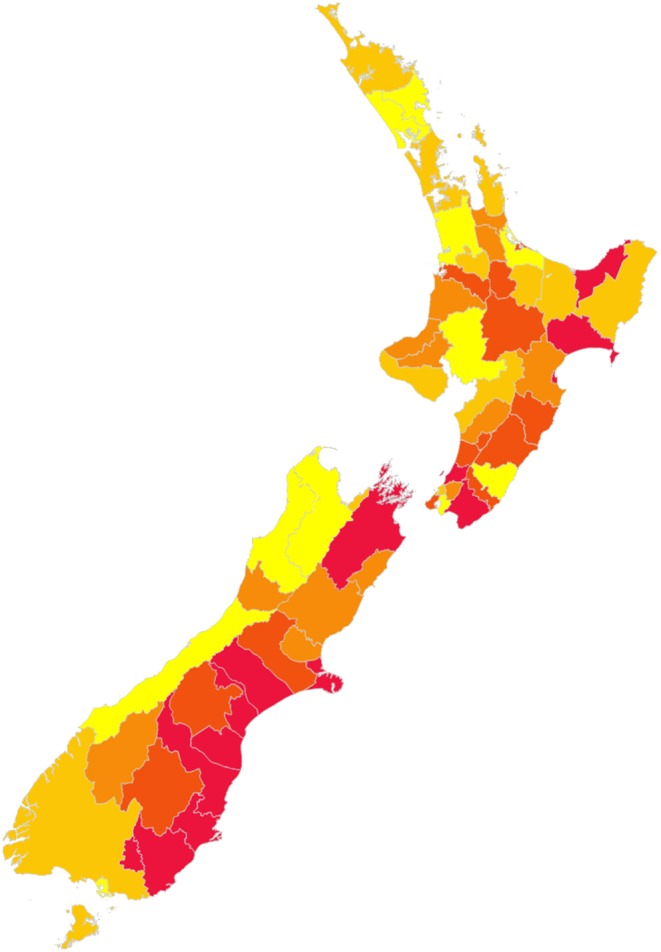
Best identified sequence of control for New Zealand, to minimize disruption of animal movements. Red indicates the first districts, yellow the last districts in the sequence.

The best sequence observed is illustrated in Figure 7.

These two examples demonstrate that, using a random sample, it is feasible to estimate a good eradication sequence that is close to the optimal, even when the number of units is large. It also shows that the movement structure has an influence on the benefits that can be gained from this approach. Where movements are largely asymmetrical, the benefits can be very large, but when most population units have both significant inward and outward units, the benefits are less marked. The scale may also play a role. What may well appear to be a source-sink dynamic at a larger scale, may not be at a smaller scale, and this should be taken into account when determining the appropriate unit size.

The optimal eradication sequence may need to take further factors into account, including seasonal variations in movement patterns, logistics and resourcing, natural geographic barriers, cultural factors etc. These may be able to be included as constraints in a dynamic programming optimization model.

#### Alternative Sequencing Approach

Detailed modeling and optimization depends on accurate animal movement data for every node. As discussed in section Surveillance, surveillance approaches exist that mean that it is feasible to capture this level of data in most target countries for PPR eradication. However, if such detailed movement data is not available, an alternative approach is available to optimize the sequence of eradication to minimize the impact of movement restrictions. Market price information systems exist in many countries and are feasible to establish where not already available (33). It is possible to use data on market prices to predict livestock movement patterns (34).

**Figure 8:**
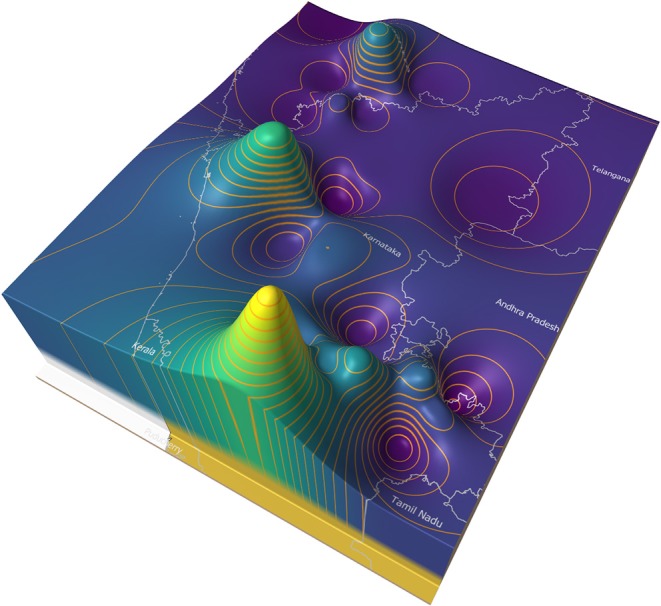
Average goat price surface for Karnataka state in 2018 with 500 rupee contours.

Figure 8 illustrates an example of a price surface plot for the average daily modal live goat market price for 2018 in and around Karnataka state, India (35). Markets were georeferenced using public sources and the price surface interpolated using an inverse distance weighted algorithm (36). Contour lines were generated at 500 rupee intervals (37). The peaks represent areas of high prices, and the troughs low prices.

In general, market forces dictate that animals are more likely to move from the lower to the higher areas. If eradication is commenced in the lowest areas (the areas where supply is highest and demand is lowest), and progresses upwards (imagine flooding the valleys), it is likely that this approach will approximate a control sequence that has a relatively low impact on trade patterns.

#### Addressing Counter-Current Movements

Regardless of the approach taken, optimizing the sequence of disease eradication is unlikely to ever result in a situation where no movements need to be blocked. The risk of producers and traders circumventing movement restrictions may be further managed by the implementation of risk-based strategies to specifically address the small number of remaining movements that would otherwise be blocked. Detailed movement data will allow risk to be assessed in detail, taking multiple factors into account, including the age, sex, and purpose of the animal, production system, and nature of the movement and the nature of the destination (slaughter, market, breeding, fattening). Risk management strategies may include direct compensation, creation of short-term alternative markets (to artificially shift the price-driven movement gradient), or risk-based measures such as allowing movement after vaccination and/or quarantine, although any such strategies should be carefully examined to ensure that it does not have counterproductive or unexpected effect on the movement network. In any case, ongoing detailed surveillance of movement patterns is needed to detect and respond to unexpected changes in a highly dynamic system.

### Biosecurity

With PPR, the main method of transmission of the virus is through direct contact between animals. Fomite spread may occur, so precautions must be taken to address this risk. One of the advantages of an eradication strategy based on intensive short-term interventions in small population units, and with a major focus on stakeholder engagement is that there is increased opportunity to work with livestock owners and herders to develop practical and effective biosecurity measures.

### Managing the Risk of Reintroduction of Infection

The strategy of rapid small-area eradication, moving quickly to other population units has the potential to eliminate PPRV after only a single vaccination round. However, after eradication is successful, the level of immunity in the population will rapidly decrease. Immunity in vaccinated animals is likely to be effectively life-long, however the rapid population turnover in sheep and goat populations means that the proportion of vaccinated animals may fall by as much as 25% per year, or even faster if susceptible animals are introduced into the population.

The movement restrictions discussed in sections Theory of Animal Movement Management and Biosecurity are intended to prevent reintroduction of infection into free populations. Naturally, in any eradication program, measures must be taken to manage these risks, but it is very unlikely that such efforts will be 100% effective.

There are two main options to deal with this residual risk of reintroduction of disease into free areas. The first (the traditional trench warfare approach) is to continue to vaccinate the free population, so that if virus is introduced, it will not spread. The problems with this approach are that it is expensive; absorbs a lot of resources that would be better spent eradicating the disease from known infected areas; must be carried on for a prolonged period; is unlikely to consistently achieve very high levels of coverage, so may allow low levels of virus circulation; and masks clinical signs making rapid detection of outbreaks much more difficult (depending on serological surveys and the use of DIVA vaccines, or antigen detection tests).

The second alternative (guerrilla warfare) is to welcome the loss of vaccine induced immunity. A non-immune population risks becoming infected and allowing disease to spread rapidly. However, it also means that, if introduced, the disease is much more likely to show easily detectable clinical signs. Clinical surveillance for early detection of new outbreaks in a non-immune population is cheaper, faster and more sensitive than using periodic surveys in a vaccinated population. However, there are two prerequisites for this approach to be successful: it requires an effective farmer-based early detection surveillance system (with near-census participation and rapid communication of suspected outbreaks—see section Surveillance); and an effective rapid response capacity for investigating and eradicating outbreaks (be it by quarantine, vaccination or stamping out). The challenge of implementing these two prerequisites should not be underestimated. However, the task is feasible, as both have previously been successfully implemented in low- and middle-income countries. Rapid response capacity is largely a resource allocation decision: do we invest resources in trench warfare, with long term vaccination of a large part of the population, or do we use those same resources for rapid response to suspected outbreaks?

### Surveillance

The proposed approach depends on timely access to complete and high quality information: an understanding of the current disease distribution, detailed information on animal movement patterns, availability of resources including personnel, transport, vaccine, market prices, and so on. The various programs to eradicate rinderpest developed surveillance and information management systems to support the effort, but these have often proven to be unsustainable (38).

The key characteristics of an effective surveillance system to support global PPR eradication (39–42) include:

- Real time data capture, with automated analysis and reporting to all relevant stakeholders
- Census-level information with complete population coverage
- High quality, reliable, clean data
- Fully disaggregated data capture
- Integrated across many data types (disease, vaccination, movement, prices etc.)
- Affordable
- Sustainable.

During Rinderpest eradication, the development of participatory epidemiology techniques (17) successfully addressed a number of these criteria. Advances in information and communication technology, cloud computing, as well as communication networks in low- and middle-income countries have all meant that solutions are now available to address the data management and communication challenges. However, technology is not able to address issues of sustainability and achieving complete population coverage. Hutchison et al. (4) describe a user-focused surveillance philosophy, applied successfully in Indonesia (29), which is based on providing an *information service* to field users. The aim is to provide immediate significant individual benefit to those that generate the data, so that they participate in the surveillance system out of self-interest, rather than compulsion. This approach has the potential to generate detailed, high quality census-level data in real time, sustainably and affordably, meeting all the requirements of PPR eradication. One key element of the approach is that it should not be focused on a specific disease. Instead, it should meet the full range of stakeholders' needs. In this way, such as system can support PPR eradication in the short term, but remain as a comprehensive and effective animal health information system long after PPR has been successfully eliminated (see section Sustainability and Multiple Utility). Such surveillance approaches, especially when coupled with rapid diagnostics, may contribute to the control of diseases such as capripox, contagious caprine pleuropneumonia and foot and mouth disease, as well as providing syndromic data to support early detection of emerging diseases.

### International Collaboration

International collaboration is necessary for successful global eradication (9). Animal movement pathways in areas affected by PPR regularly cross international borders. A rapid, effective and affordable eradication strategy, such as that proposed in this paper, depends on a closely coordinated sequence of eradication, surveillance and movement control to achieve a single global program. If animal movement pathways extend between countries, the eradication strategy must as well. Collaboration in disease eradication should include coordination of activities, which requires sharing of information.

Experience from rinderpest eradication and other disease control programs has shown that international coordination is difficult to achieve, but is possible, and is a prerequisite for successful eradication (9, 43).

### Sustainability and Multiple Utility

Sustainability is an important characteristic of disease control programs, from two perspectives. Firstly, the program has to be sustainable enough to achieve its primary goal of eradication. Lack of sustainable funding, field operational or stakeholder support can result in prematurely stopping the program, potentially eroding progress made to that point (as happened in 1979 with rinderpest eradication).

Secondly, budget decision-makers (whether national or international) rightly perceive the funding required for global eradication of PPR to be a major investment for a single-disease outcome. During rinderpest eradication, there was a great deal of rhetoric about capacity development in areas such as laboratories, epidemiological skills, surveillance and information systems, and coordination. While there have been a number of long-lasting benefits in participating countries, all too often many of these systems and capabilities, developed specifically to combat rinderpest, have proven to be unsustainable. While the prime objective was eventually achieved, many of the secondary benefits promised to donors and decision-makers have vanished or are seriously deteriorated.

**Figure 9:**
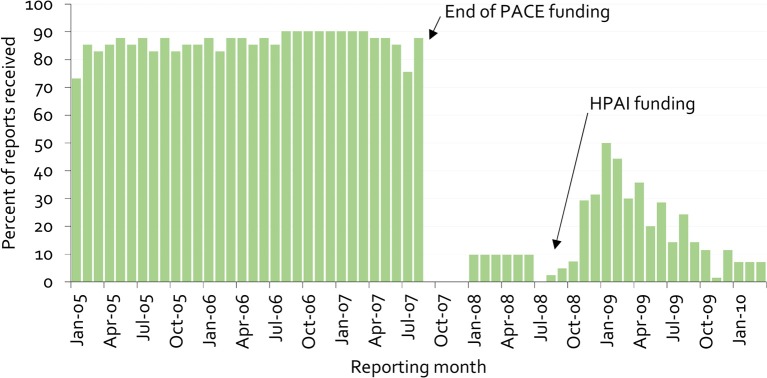
Percentage of monthly disease surveillance field activity reports for Cameroon submitted to the national authorities from 2005 to 2009 (38).

Figure 9 provides an example of this lack of sustainability, as well as clues on overcoming it. The figure illustrates the proportion of mandatory monthly field office reports received at the national veterinary office in Cameroon from 2005 to 2009 (38).

Under PACE, field officers received payment for submission of reports. When these payments stopped, the previous reporting rate of almost 90% immediately dropped to zero. The psychology behind this is simple. Payment for information gives information providers a clear message that there is no personal benefit in generating the information beyond receiving payment. More importantly, when a new program was started for highly pathogenic avian influenza in 2008, again based on payment for information, stakeholders' trust had already been eroded, and it was no longer possible to achieve high reporting rates.

**Figure 10:**
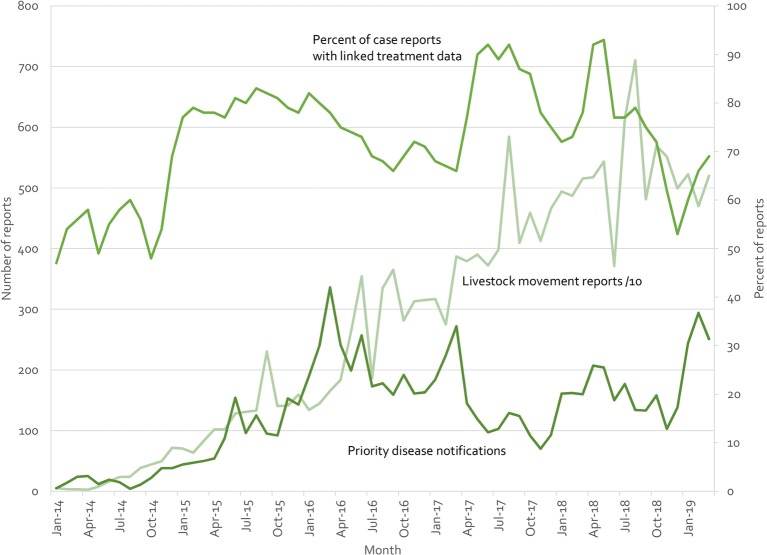
Summary of iSIKHNAS reporting from January 2014 to March 2019 for 3 of the 30 modules: Priority disease reports (number of reports, left axis), livestock movement reports (number of reports divided by 10, left axis), and treatment reports (proportion of routine disease reports with linked treatment data, percentage, right axis).

Indonesia's iSIKHNAS (4, 29) provides a counter example. Built on the a user-focused philosophy, field officers and farmers are not paid for submitting data. Instead, the system is designed to meet their daily needs for information and to make their work easier. Figure 10 presents real-time reports received by the system for three modules (treatment reports, livestock movement, and suspect priority disease notifications). There is no regulatory requirement to submit reports on animal treatments—field officers use this system because they want to, not because they have to. Yet between 50 and 90% of clinical cases are accompanied by treatment data (which may reflect the proportion of cases that require treatment, implying a near 100% reporting rate). Similarly, only 71,457 of the 190,536 movement reports (37.5%) are required by regulation (ruminants and groups of over 100 poultry). The rest are being voluntarily registered by owners and veterinary staff because of perceived benefits.

Donors and budget holders are likely to be hesitant to fund a large program that has no residual benefits for participating countries after PPR is eradicated, and they are also becoming less likely to accept claims of sustainability at face value. To gain their support, it is necessary to demonstrate value for money, both in the short term (PPR eradication) and the long term (sustainable improvement of veterinary service capacity to deal with other important disease problems).

Some of the key lessons regarding sustainability from Indonesia's iSIKHNAS include:

- **User-focused design**: the system is first and foremost designed to meet the needs of field users, not central decision-makers.
- **Capacity to manage any disease**: the system does not focus on a single disease, and can capture information on any disease of relevance to farmers and field officers.
- **Do not pay for information**: Payment for information undermines user perceptions of the value of the information, erodes data quality, and threatens sustainability.
- **Integrated information management**: integration of multiple data types (disease, population, movement, vaccination, disease control activities etc.) increases the value of the data and the power of analysis.
- **Capacity to rapidly evolve**: Disease evolves rapidly, as do stakeholders' needs and priorities. The information system needs to evolve rapidly to continue to meet their needs.

Some of the challenges faced in implementing the approach in Indonesia include:

- **Human resources**: developing and maintaining a dedicated management team.
- **Maintaining the principles**: flexibility means that new modules are being added regularly. There is a risk that stakeholders may stray from the core principle of focusing on user needs, and revert to top-down approaches, undermining sustainability.
- **Meeting user demands**: user expectations for immediate access to customized analyses are high, placing a strain on the management team to constantly deliver.
- **Heterogenous user groups**: it is difficult to achieve 100% participation due to variability in stakeholders perceptions of benefits, the system managers' capacity to understand these perceptions, and their ability to deliver the breadth of customized outputs to meet their needs.

## Phased Implementation

The previous section provided the theoretical background for the proposed PRR eradication strategy. These components need to be combined in a coordinated way to achieve the objective of rapid, affordable eradication.

A four-phase approach is proposed. This is broadly compatible with the Global Strategy (2), a step wise approach that stresses the importance of progressive and epidemiologically sound activities. This discussion intentionally omits a lot of the important detail contained in that document. The Global Strategy is built around the concept of progressive development of national capacity. Under the guerrilla approach, there should be less emphasis on national boundaries, and more emphasis on a coordinated single program. The proposed phases are:

1. Building the required foundations—funding, coordination, engagement and information
2. Detailed planning
3. Implementing eradication activities
4. Demonstrating successful eradication.

### Phase 1: Foundations

It is proposed that a large part of the time spent on eradication should be spent on laying strong foundations for the program, before any specific eradication activity starts. During this phase, existing disease control activities may be continued, but their purpose is simply to prevent further spread and limit losses, not to start eradication. As with the global program, it is anticipated that this phase may last several years. It will required major investment, particularly to develop stakeholder engagement, surveillance and information systems.

#### Funding

There is no point in embarking on global eradication if there are not enough resources to complete the task. As was seen with rinderpest eradication, stopping too soon, and withdrawing funding can mean a delay of years, a loss of millions of animals, and dramatically increase the total cost of the program. Many (but not all) currently affected countries are unlikely to have the resources to fund eradication from their own national budgets. Eradication of PPR should be considered a global public good, so there is a strong justification for multilateral donors to fund a large proportion of the program. Jones et al. (3) have provide estimates of the benefits and costs of PPR eradication, and the process of securing these funds has already started. The guerrilla approach may make the program less expensive, but securing funds will take time and should be among the first priorities.

#### Coordination

Close international coordination also requires some time to establish. Planning the most effective strategy for eradication requires very good surveillance information from all participating countries. Coordination is required from the outset so that all countries involved are able to improve their surveillance and generate the information required for effective planning.

#### Engagement

A key feature of the proposed guerrilla strategy is the need for close and ongoing farmer and broader community engagement. Detailed surveillance information, achieving very high levels of vaccination coverage, effective movement management, and highly sensitive early detection and response to new outbreaks in free areas, all depend on strong stakeholder participation in the eradication program. Locally appropriate approaches to building this engagement are required, but adherence to a simple core principle has been found to be effective (4): ensuring that participation provides significantly more direct, immediate, personal benefits than any costs or risks associated with the program.

#### Information

Successful implementation of this strategy depends on detailed, high quality information. Distinguishing features include: dividing the population into small groups; aiming for a short period of high vaccination coverage; highly strategic movement management; and highly sensitive early detection and response—but none of these are possible without detailed information.

The information requirements for effective implementation are extremely demanding and include:

- A complete knowledge of the population including, animal numbers, husbandry, spatial distribution, and reproductive, marketing and slaughter patterns
- An initial understanding of the distribution of PPR in the population, to define population units that are already free from disease
- An understanding of available resources for vaccination, surveillance, movement management and emergency response
- Information on the immune status of population units and individuals
- Detailed flock-level information on animal movements, and an understanding of the sociological and economic drivers for movements
- Accurate tracking of control activities including vaccination, biosecurity, checkpoints and outbreak response
- Extremely sensitive early detection surveillance to identify and respond to outbreaks in free areas.

Capturing this data requires census-level participation of farmers, field disease control personnel, extension officers and the broader farmer support networks. It requires fit-for-purpose communication tools and powerful integrated data management capacity. None of this is easy, but the Indonesian iSIKHNAS system (4, 29) provides a model of how it can be achieved. User-focused surveillance and production information systems have the benefit that they address the broader needs and interests of farmers and other field stakeholders, which go well beyond the requirements of the eradication of a single disease. As a result, a system that is developed to support PPR eradication may be used to sustainably support the control of a full range of other diseases, now and in the future.

Implementation of high-coverage, user-focused systems is complex, requiring a blend of sociological, epidemiological and information and communication technology skills. However, with adequate resources, based on experience of implementation in Indonesia, it may be able to be achieved within about 3 years.

In addition to information provided by surveillance and production information systems, there may be specific research questions that need to be addressed. One obvious example is whether the guerrilla strategy proposed in this paper can actually work. Pilot studies may be used to answer this and other questions (2).

### Phase 2: Planning

This phase involves using the information gathered to develop a detailed comprehensive and integrated plan for eradication. It involves [in addition to elements already identified in the Global Strategy (2)] delimiting the distribution of the disease and identifying non-infected populations; defining suitable population units; analyzing movements to minimize trade disruption during eradication; planning the logistics of disease eradication including vaccination, movement management, and rapid response teams.

### Phase 3: Implementation

Active eradication efforts should only start when all the requirements are in place, including funding, coordination, detailed information supporting detailed plans, trained and resourced field teams, and strong stakeholder support.

Eradication involves rapidly moving through all infected population units in the optimal sequence to minimize trade disruption. In each unit, available resources are concentrated for a single vaccination round, to achieve a very high, rapid coverage, and maintain the coverage for approximate 1 month. Livestock owner participation will have already been strengthened during the foundational phase, but will be further enhanced prior to the initiation of vaccination. During this control phase, the only inward movements permitted are of vaccinated animals from free areas. Outward movements to infected areas are possible.

After the control period, the population unit is considered to be free for the purposes of movement management: incoming movements from free units are permitted, as are outgoing movements to infected units. Farmer-based intensive clinical surveillance is used to support early detection of any new incursions, and rapid response teams are available to investigate, isolate and eradicate any new outbreaks in free units. The population units used for control can be progressively aggregated into free and control zones, as defined in the Global Strategy.

High levels of vaccination coverage and good clinical surveillance are supported by engaged farmers, and well trained and resourced vaccination and emergency response teams. If farmer support is inadequate to ensure high coverage or good surveillance, then the preparatory work on stakeholder engagement has not been adequate, and new, more effective approaches need to be adopted.

### Phase 4: Demonstration of Global Freedom

The last case of smallpox occurred in 1977 in Somalia and it was declared eradicated 3 years later in 1980. The last confirmed case of rinderpest occurred in 2001 (also in Somalia), and global eradication was declared in 2011, after a major surveillance effort. Confirming global eradication is a critical step and we can't afford to get it wrong. For PPR, the eradication strategy depends on the presence of highly sensitive, sustainable, multi-disease early detection systems. Martin et al. (44–46) and Cameron (47) show how confidence in freedom from infection can be quantified and accumulate with increasing evidence, a method that was also applied to demonstrate global freedom from rinderpest (48). It is anticipated that using such systems, the declaration of freedom from PPR could be made much sooner than the 10 years it took to build global confidence of freedom from rinderpest.

## Discussion

The trench warfare approach to rinderpest eradication used vaccination as its main weapon, seeking to maintain herd immunity in large populations for extended periods. Lengthy campaigns resulted in a gradual erosion of support by funders, field staff and farmers. The result was a 50 year war, that was only finally won when a more strategic approach was adopted.

When adopting guerrilla tactics, the main weapon is information and the application of epidemiological understanding of PPR. The key strategy to accessing information is the use of sociological approaches to working with field stakeholders, to build strong and sustainable engagement, and to convincingly answer their question: “What's in it for me?” Vaccination, while still an essential tool, should play a relatively much smaller role in eradication, while intelligent risk-based movement management, highly sensitivity early detection and rapid and effective outbreak response are all critical components.

### Challenges and Risks

This paper has presented a hypothesis—that the guerilla approach is able to support global eradication of PPR more quickly and less expensively than the trench warfare approach. The characteristics and advantages of the guerilla approach have already been discussed. However, challenges and risks warrant consideration.

The success of the approach depends on a range of assumptions. Related to stakeholder engagement, surveillance and information, these assumptions include that:

- It is possible to use sociological and other related approaches to understand field stakeholders' needs and motivations, and to develop systems that provide meaningful immediate direct benefit, adequate to ensure sustainable, widespread support of disease eradication activities.
- Using this approach, it is possible to achieve very high coverage surveillance.
- Communication and information management technologies are suitable and available to support real-time high-volume disaggregated data capture and analysis.

Related to vaccination, they include:

- It is possible to achieve very high vaccination coverage in small population units.
- High vaccination coverage will result in high levels of protection (implying that vaccine quality, transport, vaccination technique and the ability of animals to mount an immune response are all good).
- Achieving high levels of protection for a short period (for example, 5 weeks) in a small closed population will be effective at eliminating the virus.

Related to movement management, they include:

- It is possible to largely prevent the spread of PPR from infected and/or control units to free units through epidemiologically informed management of animal movements.
- A strategic approach to movement management that minimizes disruption to trade is possible.
- It is feasible (and preferable) to implement rapidly changing, short duration movement restrictions.

Related to early detection and response, the assumptions include:

- Population turnover and movement in free areas will result in a rapid drop in the proportion of protected animals (to levels below that required for herd immunity) within a year.
- New outbreaks of PPR in free areas will exhibit readily identifiable clinical signs.
- It is possible to implement a highly sensitive farmer-based early warning system, including the communication tools required for rapid notification.
- The veterinary services have the capacity to mount a rapid response to notifications for diagnosis and, where required, local eradication (either by vaccination and movement management, or stamping out).

A number of these assumptions have been demonstrated to be invalid at various stages of rinderpest eradication (11). On the other hand, the world learnt many lessons from rinderpest, and advances in the integration of sociological techniques into disease control, as well as information and communication technologies mean that some of the challenges may now be able to be successfully addressed.

### Wider Application

The strategy presented in this paper focuses on PPR eradication. It is worth considering whether the same approach may be applicable to local, national or global eradication of other diseases.

The characteristics of PPR (fragile virus with short survival outside the host, life-long immunity after a single vaccination, main method of transmission by direct contact, lack of significant reservoirs or carrier state) mean that the guerrilla approach may be well suited to this virus. Other diseases are clearly not suitable. For example, this approach would not be relevant to African Swine Fever (ASF), with no vaccine, lengthy survival outside the host, the existence of intermediate or reservoir hosts (ticks) and the potential for carrier states. Without vaccination, strict biosecurity, movement management and stamping out are the main control options available. Nevertheless, information on disease distribution, early detection and risk pathways is still critically important.

Foot and mouth disease (FMD) represents an intermediate example—not as amenable to eradication as PPR but potentially easier than ASF. In this case, a vaccine exists, but it does not provide long-lasting immunity. The virus is more resistant and infects more species, as well as being able to be transmitted by fomites, animal products as well as through airborne spread (in specific conditions). Vaccination and movement management have long been the important tools for FMD control, and it is possible that the guerrilla approach may make a useful contribution with this disease. The shorter duration of immunity after vaccination is not a problem, if effective clinical surveillance is able to detect subsequent outbreaks. However, the approach would have to be adapted, with an even greater emphasis on biosecurity, including preventing spread via fomites and animal products.

### Potential Contribution

Table 3 summarizes the way in which the various aspects of the guerrilla strategy may be able to address the key challenges of rapid, affordable, effective PPR eradication.

**Table 3:** Selection of key challenges facing global PPR eradication, and an indication of how different components of the guerrilla strategy (phases 1–3) may be able to address them (see footnotes for clarification of the challenges and components).

	**Phase 1: Foundation**							**Phase 2: Planning**			**Phase 3: Implementation**			
	**Farmer engagement**	**User-focused surveillance^**a**^**	**Information system^**b**^**	**Information^**c**^**	**International coordination**	**Assured funding^**d**^**	**Laboratory** **and** **vaccine^**e**^**	**Strategic implementation^**f**^**	**Comms and engagement**	**Resources** **and** **logistics**	**Small units^**g**^**	**Rapid progression^**h**^**	**Farmer reporting^**i**^**	**Rapid response^**j**^**
**ACHIEVING VERY HIGH LEVELS OF PROTECTION**														
**Vaccination coverage**														
Inadequate communication with owners	•	•		•					•	•	•			
Physical access to flocks	•			•	•			•	•	•	•			
Owner compliance	•	•	•						•		•	•		
Inadequate supply of vaccine				•	•	•		•		•	•			
Inadequate time/human resources														
Corruption within vaccination teams	•		•	•						•	•			
**Vaccination rate**														
Time required to vaccinate the population	•			•				•		•	•			
**Immune response**														
Choice of vaccine				•			•							
Poor quality control and potency				•			•							
Poor handling and cold chain										•	•	•		
Poor vaccination technique										•	•			
*Poor immune response*														
Stress, malnutrition, concurrent disease etc.^k^	•	•	•	•				•			•			
Maternal immunity	•	•	•	•				•			•			
**Reproduction**														
New lambs/kids diluting immune population		•	•	•				•			•	•		
**Movement**														
Movement from infected areas	•	•	•	•	•			•	•		•	•		
Movement from free areas diluting immunity	•	•	•	•	•			•	•		•	•		
**MOVEMENT MANAGEMENT**														
Understanding movement pathways	•	•	•	•	•									
Flock or animal identification	•		•		•				•	•				
Market distortion due to movement management	•			•				•			•	•		
Farmer non-compliance with movement restrictions	•			•				•	•		•	•		
Transhumant or migratory production systems	•			•				•	•		•	•		
Cross-border movement patterns	•			•	•			•	•		•	•		
Rapid changes in movement patterns	•	•	•	•	•			•	•		•	•		
**EARLY WARNING SURVEILLANCE**														
Low disease reporting rates	•	•	•						•				•	
Low farmer awareness	•	•							•				•	
Poor field communication	•		•						•				•	
Fear of negative consequences for reporting	•	•							•				•	
Lack of veterinary field surveillance resources	•	•	•			•				•			•	•
**RAPID OUTBREAK RESPONSE**														
Delay in receiving field reports of suspect outbreaks	•	•	•						•				•	
Inadequate capacity for rapid field investigation						•				•				•
Inadequate laboratory diagnostic support							•							
**SUSTAINABILITY**														
Lack of sustainability of systems developed for PPR eradication	•	•	•						•					

a User-focused surveillance: a surveillance systems designed around users' needs, capturing data on all diseases of significance to farmers, and designed to maximize direct user benefits while eliminating any costs or risks associated with participation.

b Information system: a real-time integrated health and production information system, with field mobile data capture, automated analysis and alerts, managing (at least) disease reports, animal/flock identification, vaccination, control activities, movement management, and emergency response.

c Information: detailed, real-time information on animal populations, animal movements, disease distribution, immune status, resources and capacity, vaccination and disease control activities.

d Funding: Coordinated, adequate and sustained funding through international donors and national budgets.

e Laboratory and vaccine: Adequate laboratory diagnostic and monitoring capacity and quality control, and access to high quality adequate vaccine supplies (these issues have been well-addressed in existing PPR eradication strategies).

f Strategic implementation: Technical analysis of the appropriately sized and delimited population units, the sequence and timing of eradication.

g Small units: Definition of small population units as the building blocks for eradication, where available resources can be concentrated to achieve very high coverage, high quality vaccination for disease elimination.

h Rapid progression: Rapidly moving in an optimal sequence through all infected population units eliminating the virus and managing movement.

i Farmer reporting: an effective, highly sensitive and timely farmer-based early warning reporting system, built on effective farmer engagement and the user-focused surveillance, to achieve rapid reporting of all suspect disease events.

j Rapid response: rapid response capacity within the veterinary services working in partnership with local communities, including mobile investigation and response teams, pen-side and laboratory diagnostic capacity, and appropriate local response strategies (vaccination, stamping out, quarantine etc. as required).

k *Poor immune response: Of the various reasons for failure to achieve high flock immunity, stress, malnutrition, concurrent disease and similar problems are among the most difficult to address. Working with farmers to minimize these conditions, and optimizing timing may help*.

## Conclusion

Global PRR eradication is a grand project, requiring vision and innovation. It is hoped that elements of the guerrilla hypothesis presented here may be tested, and ultimately contribute to finding an affordable way to rapidly achieve this goal.

## Data Availability Statement

Two of the datasets analyzed for this study (32, 35) are publicly available at the URLs provided in their citations. The Java goat movement dataset is not publicly available as it has been sourced from an internal government database. Requests for access to the data should be directed to Muhammad Muharam Hidayat, mm.hidayat.andi@gmail.com.

## Author Contributions

AC developed the hypothesis, designed and undertook the analysis, and drafted and revised this paper.

### Conflict of Interest

AC was employed by Ausvet Europe.
